# ADVERSE CUTANEOUS DRUG REACTION

**DOI:** 10.4103/0019-5154.39732

**Published:** 2008

**Authors:** Surajit Nayak, Basanti Acharjya

**Affiliations:** *From Department of Skin and VD, MKCG Medical College and Hospital, Berhampur, Orissa, India*

**Keywords:** *ACDR*, *adverse drug reactions*, *Steven Johnson syndrome*, *toxic epidermal necrolysis*

## Abstract

In everyday clinical practice, almost all physicians come across many instances of suspected adverse cutaneous drug reactions (ACDR) in different forms. Although such cutaneous reactions are common, comprehensive information regarding their incidence, severity and ultimate health effects are often not available as many cases go unreported. It is also a fact that in the present world, almost everyday a new drug enters market; therefore, a chance of a new drug reaction manifesting somewhere in some form in any corner of world is unknown or unreported. Although many a times, presentation is too trivial and benign, the early identification of the condition and identifying the culprit drug and omit it at earliest holds the keystone in management and prevention of a more severe drug rash. Therefore, not only the dermatologists, but all practicing physicians should be familiar with these conditions to diagnose them early and to be prepared to handle them adequately. However, we all know it is most challenging and practically difficult when patient is on multiple medicines because of myriad clinical symptoms, poorly understood multiple mechanisms of drug-host interaction, relative paucity of laboratory testing that is available for any definitive and confirmatory drug-specific testing. Therefore, in practice, the diagnosis of ACDR is purely based on clinical judgment. In this discussion, we will be primarily focusing on pathomechanism and approach to reach a diagnosis, which is the vital pillar to manage any case of ACDR.

## Definition and Classification

An adverse cutaneous reaction caused by a drug is any undesirable change in the structure or function of the skin, its appendages or mucous membranes and it encompass all adverse events related to drug eruption, regardless of the etiology.

Drug reactions can be classified into immunologic and nonimmunologic etiologies [Table 1]. The majority (75-80%) of adverse drug reactions are caused by predictable, non-immunologic effects, the remaining 20-25% of adverse drug events are caused by unpredictable effects that may or may not be immune-mediated.^1^ Immune-mediated reactions account for 5-10% of all drug reactions and constitute drug allergies falling into this category.^2^,^3^

**Table 1:** Immunologic and nonimmunologic drug reactions

Immunologic	Nonimmunologic
**Type I reaction (IgE-mediated)** Anaphylaxis from lactam antibiotic	**Predictable**
	**Pharmacologic side effect** Dry mouth from antihistamines
**Type II reaction (cytotoxic)** Hemolytic anemia from penicillin	**Secondary pharmacologic side effects** Thrush while taking antibiotics
**Type III reaction (immune complex)** Serum sickness from antithymocyte globulin	**Drug toxicity** Hepatotoxicity from methotrexate
	**Drug-drug interactions** Seizure from theophylline while taking erythromycin
**Type IV reaction (delayed, cell-mediated)** Contact dermatitis from topical antihistamine	**Drug overdose** Seizure from excessive lidocaine (Xylocaine)
**Specific T-cell activation** Morbilliform rash from sulfonamides	**Unpredictable**
**Fas/Fas ligand-induced apoptosis** Stevens-Johnson syndrome Toxic epidermal necrolysis	**Pseudoallergic** Anaphylactoid reaction after radiocontrast media
	**Idiosyncratic** Hemolytic anemia in a patient with G6PD deficiency after primaquine therapy
**Other** Drug-induced, lupus-like syndrome Anticonvulsant hypersensitivity syndrome	**Intolerance** Tinnitus after a single, small dose of aspirin

G6PD - glucose-6-phosphate dehydrogenase

Another classification is Gell and Coombs classification system that describes the predominant immune mechanisms that lead to clinical symptoms of drug hypersensitivity. However, some drug hypersensitivity reactions are difficult to classify because of lack of evidence supporting a predominant immunologic mechanism. These include certain cutaneous drug reactions (i.e., maculopapular rashes, erythroderma, exfoliative dermatitis and fixed drug reactions)^4^,^5^ and specific drug hypersensitivity syndromes.

## Prevalence

Adverse cutaneous reactions to drugs are frequent, affecting 2-3% of all hospitalized patients. Fortunately, only approximately 2% of adverse cutaneous reactions are severe and very few are fatal. Unfortunately, there are few recent data on the epidemiology of ADRs.^6^ The incidence of ACDR in developed countries range from 1 to 3% among in patients,^7^,^8^ whereas in developing countries such as India, some studies peg it to 2-5% of the in patients;^9^-^12^ however, there is a lack of comprehensive data amongst out-patients. The inadequacy of data could be attributed to reasons such as diagnostic dilemmas and lack of awareness to report. In the United States, more than 100,000 deaths are annually attributed to serious adverse drug reactions.^13^ In a study conducted by Chatterjee *et al.* the incidence of drug-induced adverse skin reactions is found to be 2.6% at a dermatology outpatient setting.^14^ The incidence of adverse reactions increases up to 15.1% when both serious and nonserious reactions are calculated.^13^ Cutaneous reactions comprise approximately 2-3% of all adverse drug reactions^15^ and approximately 1 in 1,000 hospitalized patients has a severe cutaneous drug reaction.^16^ In a study examining the incidence of skin eruptions, approximately 45% of all the adverse drug reactions were manifested in the skin.^17^ Cutaneous drug reactions occur in 2-3% of all hospitalized patients. Most drug eruptions are mild, self-limited and usually resolve after the offending agent has been discontinued. Severe and potentially life-threatening eruptions occur in approximately 1 in 1000 hospital patients. Mortality rates for erythema multiforme (EM) major are significantly higher. Stevens-Johnson syndrome (SJS) has a mortality rate below 5%, whereas the rate for toxic epidermal necrolysis (TEN) approaches 20-30% and most patients die from sepsis. The incidence of adverse cutaneous reactions to drugs is higher in women than in men, and elderly patients have an increased incidence of adverse drug reactions.

The analysis of the data by Chatterjee *et al*.^14^ shows that urticaria and fixed drug rashes were the most common morphological reaction-types. Carbamazepine and phenytoin were the most common offenders. The common offending drug groups as stated by the same group were antimicrobials (34.10%), anticonvulsants (32.88%), anti-inflammatory drugs (21.51%). Other less frequent ones were antipsychotics, antidepressants, antihypertensives oral contraceptives, antidiabetics, insulin, vaccines, radio contrasts, pancreatic enzyme supplements, homeopathic and ayurvedic preparations. The most common offending drugs were carbamazepine (16.23%), phenytoin (15.15%) and cotrimoxazole (13.53%); however, antimicrobials were the most common drug group implicated. Various studies shows that most common morphologic patterns are exanthematous, urticarial and/or angioedema, fixed drug eruption and erythema multiforme.^18^ Others have also noted exanthematous eruption to be the most common type of drug eruption.^19^,^20^ Exanthematous drug eruptions, also known as maculopapular drug eruptions, are the most common cutaneous skin reactions and represent approximately 95% of all cutaneous drug eruptions.^7^ Exanthematous drug eruptions usually begin within 1-2 weeks of starting a medication and gradually resolve 1-2 weeks following cessation. A study from North India also found maculopapular rash to be the most common type of ACDR.^21^ In a study by Thappa *et al.*^22^ most common eruptions observed were fixed drug eruptions (31.1%) and maculopapular rash (12.2%). This variation could be due to different patterns of drug usage and different ethnic group characteristics.

## Factors

Certain patient groups [Table 2] appear to be at an increased risk of developing a cutaneous drug reaction. The incidence of cutaneous reaction increases with the number of drugs taken.^7^ In addition, some drug interactions may contribute to the development of a skin eruption. The age of the patient also correlates with an increased risk of a cutaneous drug eruption; older patients, boys younger than 3 years and girls older than 9 years have been found to be more prone to skin drug eruptions. Women are more likely than men to develop a skin drug eruption. Viral infections have also been shown to increase the risk of a drug rash. Some intrinsic factors influence the risk of cutaneous drug eruptions, such as genetic variations in the metabolism of a drug and HLA association. Finally, intercurrent diseases such as systemic connective tissue disease may lead to immune perturbance and enhance the risk of a cutaneous drug eruption as impaired renal and liver functions increase the risk of cutaneous rashes. Drug factors involve the following: the route of administration (more common with topical and intramuscular administration and less so with intravenous administration; oral route is the safest^3^); duration (more common with chronic or frequent use rather than short-term or intermittent use^2^); dose and variation in metabolism.^2^ Reactions are more common for drugs with low therapeuticx indices, high levels of drug-drug interactions and a tendency to form reactive intermediates or toxins.^17^ The most importantx drug-related risk factors for drug hypersensitivity concern the chemical properties and molecular weight of the drug. Larger drugs with greater structural complexity (e.g, nonhuman proteins) are more likely to be immunogenic. Heterologous antisera, streptokinase and insulin are examples of complex antigens capable of eliciting hypersensitivity reactions. Most drugs have a smaller molecular weight (less than 1,000 Daltons), but may still become immunogenic by coupling with carrier proteins, such as albumin, to form simple chemical-carrier complexes (hapten). Even environmental factors may contribute to an adverse reaction.

**Table 2:** Patient risk factors for adverse drug reactions

General drug reactions (nonimmune)	Hypersensitivity drug reactions (immune)
Female gender	Female gender
Serious illness	Adult
Renal insufficiency	HIV infection
Liver disease	Concomitant viral infection
Polypharmacy	Previous hypersensitivity to chemically related drug
HIV infection	Asthma
Herpes infection	Use of beta blockers
Alcoholism	Specific genetic polymorphisms
Systemic lupus erythematosus	Systemic lupus erythematosus

## Approach

The most important point is to have a high level of suspicion regarding the possibility of ACDR. The next most important aspect is to identify the offending drug because once you identify it, the management is possible and easy. Any new dermatitis/rash in a patient without prior skin disease should prompt the consideration of drug sensitivity. The diagnosis can be straightforward when a patient who takes few or no medications develops a rash after starting a new drug. The challenge arises when a patient takes many medications, including a few new ones, because any one of these could cause an adverse reaction. The evaluation should begin with a drug history, including the identification of all drugs the patient has been administered in the recent past. Moreover, the clinician must understand that adverse reactions can occur as late as 2 weeks after a medication has been discontinued. The knowledge of prior allergies helps to identify any cross-reactivity to current medications.

The first step is to review the complete medication list of the patient, including over-the-counter supplements. The second step is to find out any history of previous adverse reactions to drugs or foods. Consider and exclude alternative etiologies, especially viral exanthems and bacterial infections. Initial history should include a record of all prescription and nonprescription drugs taken within the last month, including the dates of administration and dosage. Unless the patient has been previously sensitized to a drug, the interval between the initiation of therapy and the onset of reaction is rarely less than 1 week or more than 1 month. Patients should be asked about previous drug exposures and reactions. The right diagnosis of ACDR is one of the key elements in the assessment of a patient before instituting any treatment or recommendations. This diagnosis can sometimes be very difficult to establish, as a cutaneous drug eruption can closely mimic other common skin diseases. Moreover, if a patient is taking several drugs, as is often the case, the identification of the causative drug becomes considerably complex. Therefore, a rationale and organized approach is advisable. The components of this approach are the following:

## Obtaining the History

A good and complete history taking is very vital and helpful and it includes the following:

1. General medical history as indicated
2. Drug exposure (dosage, date started, duration and interruptions in use)
3. Use of proprietary remedies (e.g., herbals)
4. Initiation of drug use and onset of reaction
5. Previous adverse drug reactions, cutaneous and otherwise, and type of adverse reaction
6. Re-exposure to a drug and exacerbation of eruption
7. Improvement after a decrease in dosage or discontinuation of drug
8. Disease states or injuries that may cause the eruption or act as cofactors (e.g., infectious mononucleosis and ampicillin-related reactions or HIV and trimethoprim-sulfamethoxazole (Bactrim) reactions)
9. Previous family or personal history of skin disease
10. Family history of hypersensitivity syndromes and anticonvulsant hypersensitivity syndrome
11. Environmental/occupational exposure to other substances that may be the etiologic agents (e.g., sunlight, artificial tanning devices)

## Initial Clinical Impression

The initial clinical impression is principally based on the morphology of the cutaneous eruption. Four main categories are described on the basis of the primary lesion: exanthematous, urticarial, blistering or pustular. Extracutaneous signs (e.g., malaise, fever, hypotension, tachycardia, lymphadenopathy, synovitis, dyspnoea, etc.) allow for refinement of the primary clinical impression. In many cases, the presence of these extracutaneous signs may aid in distinguishing benign cutaneous drug eruptions from potentially severe systemic drug eruptions.

## Differential Diagnosis

Establishing a differential diagnosis is essential in order to include all possible diagnoses and avoid missing the right diagnosis. This step is based on the same criteria as the initial clinical impression (e.g., cutaneous morphology and extracutaneous signs).

## Analysis of Drug Exposure

The careful analysis of drug exposure must be undertaken in order to identify the causative drug of the suspected drug eruption. Medications of all types, whether allopathic, homeopathic, ayurvedic, natural or traditional products in any form, regardless of the route of administration must be considered, especially new drugs taken in the first 8 weeks before the skin reaction. Drugs taken intermittently or on an as-needed basis must also be considered. The temporal relationship between drug intake and the onset of clinical symptoms is critical. Unless the patient has been previously sensitized to a drug, the interval between initiation of therapy and the onset of reaction is rarely less than 1 week or more than 1 month. Patients should be asked about previous drug exposures and reactions.

Patients should be questioned regarding all vitamins, pain medications, sedatives, laxatives, oral contraceptives, over-the-counter medications and natural products. Dechallenge (improvement after a decrease in dosage or stopping of drug) and rechallenge (recurrence or exacerbation of eruption after re-exposure to a drug) are also important to document but should not be tried by a physician and is contraindicated. Drug interactions can also be noted because these can sometimes precipitate a drug rash.

## Analysis of the Literature

A literature search may provide information regarding the frequency with which the specific morphologic pattern may be related to a particular drug and association of the disease with the drug. One must be updated with textbooks of drug eruptions, literature searches, online databases and adverse drug reaction software periodically. The DERM index and MEDLINE are very useful references for the dermatologist. However, it is important to keep in mind that regardless of literature data, all drugs must be considered to be the possible cause of any reaction, even if a drug is not widely known to be associated with a particular reaction.

## Analysis of Laboratory Results and Diagnostic Tests

The goal of diagnostic testing is to evaluate biochemical or immunologic markers that confirm the activation of a particular immunopathologic pathway to explain the suspected adverse drug effect. Laboratory evaluation is guided by the suspected pathologic mechanism. Several diagnostic tests can help to identify a suspected drug allergy; however, most have limited usefulness because many allergic reactions result from drug metabolites, which cannot be detected.^3^

The following tests may be useful:

1. Skin biopsy: Cutaneous biopsies (histopathology and direct immunofluorescence) can be useful in the management of a cutaneous eruption. The presence of eosinophils, edema and inflammation all suggest hypersensitivity. Vasculitis and necrotic changes may suggest erythema multiforme, Stevens-Johnson syndrome or toxic epidermal necrolysis. They can sometimes distinguish between a drug-induced disease and other disease; for example, a toxic epidermal necrolysis (TEN) can mimic a staphylococcal scalded skin syndrome, but a cutaneous biopsy would differentiate between the two. However, these biopsies do not allow for the identification of the causative drug.
2. Laboratory tests:
  - Similar to any other disease, different laboratory tests are available in order to evaluate and confirm the diagnosis and different possible etiologic causes of the eruption. In some cases, blood workups are useful in order to aid the clinical diagnosis. These include complete blood count (atypical lymphocytosis, neutrophilia, eosinophilia, etc.) and liver and renal function tests. Various textbooks^23^ state that an elevated peripheral eosinophil counts is an uncommon finding in cutaneous drug eruptions, and, therefore, in contrast to the popular belief, its presence or absence is of little importance in excluding or confirming the diagnosis. According to Romagosa *et al.*^24^ a peripheral eosinophil count carries little diagnostic value in the setting of adverse cutaneous drug eruptions. Guidelines of the American Academy of Dermatology state that eosinophil counts more than 1000 cells/mm^3^ indicate a serious drug-induced cutaneous eruption.^25^ All other blood tests (enzymes, electrolytes, biochemistry, ESR, ANA, bacterial and viral serology, etc.) can be requested depending on the suspected diagnosis. Culture (skin, blood, tissue, etc.) and medical imaging can also be carried out if appropriate, which may aid in confirming or ruling out potential diagnoses.
  - Drug levels are of value when eruption is associated with over dosage or other nonallergic type of reaction or in a comatose or noncommunicative patient to establish the presence of drug. It can also be useful to confirm the presence of the drug at the time of the rash as well as the overdose of this drug.
  - Enzymes and metabolitesx: For immediate-type reactions, determining the tryptase level may be helpful because tryptase is a marker of mast cell degranulation. Histamine, tryptase and beta-tryptase levels have proved useful in confirming acute IgE mediated reactions, but negative results do not rule out acute allergic reactions.^26^,^27^
3. In selected cases re-exposure may be of value; however, considering the degree of drug reaction and risks, this is usually not advisable.
4. Prick or scratch test: Emergency resuscitation equipment should be available. Skin testing, including prick and intradermal testing, has been found to be useful for the confirmation of IgE-mediated immediate hypersensitivity reactions.
5. Patch test may be helpful to confirm allergic contact dermatitis, fixed drug eruptions, exanthematous drug eruptions, drug hypersensitivity syndrome, acute generalized exanthematous pustulosis (AGEP) and other delayed skin eruptions; however, no validated protocol has been established for these tests.
6. Skin culture (bacterial, viral, fungal)
7. Microscopic tests
8. Evolving diagnostic tests: If available, more specific laboratory testing for complement levels (CH50, C3, C4) or circulating immune complexes can be conducted. Positive tests help in confirming the clinical diagnosis; negative tests do not exclude the diagnosis of immune complex disease. Systemic vasculitides induced by medication may be detected by autoantibody tests such as antinuclear antibody or antihistone antibody.^28^

*In vitro* lymphocyte toxicity assay, migration inhibition factor and other laboratory tests of lymphokine production remain investigational tools that at present are insufficiently standardized to allow clinical application.

## Prioritization of Diagnoses

The final step is the establishment of the final diagnosis and it is by far the most important because this diagnosis will determine the treatment. However, it is not always possible to be sure of the correct final diagnosis. Therefore, a prioritization of the different diagnoses previously considered must be done. This prioritization can be done by the integration of all clinical and para-clinical exams reported previously (analyses of drug exposure, literature, laboratory testing, diagnostic tests, etc.) with the initial clinical impression and its differential diagnoses. The traditional approach of grading, i.e., “highly probable,” “probable,” “possible,” “unlikely” or “almost excluded” remains useful. There are decision aids available in questionnaire or computerized spreadsheet form, which may utilize a database to deal with the problems of adverse reactions, but these are not always available. However, the clinical judgment must not be discarded. Furthermore, the more severe the reaction (drug hypersensitivity syndrome, Stevens-Johnson syndrome, toxic epidermal necrolysis), the lower the threshold of the possible diagnoses in order to ensure proper recommendation and advice regarding drugs. In this manner, physicians may follow a thoughtful and comprehensive clinical approach to the diagnosis and management of adverse cutaneous drug reactions.

The approach can be followed if we cite an example:

### Example

The following case reports are examples of establishing a diagnosis in a patient having a cutaneous eruption based on the clinical approach described here.

Case 1: A 35-year-old man, a known hypertensive, with fever and cough, was admitted to the hospital for pneumonia. He developed a widespread nonfollicular pustular eruption on an erythematous base in the folds after receiving antibiotics for his condition. The lesions started on face. He received a cephalosporin (cefuroxime), a macrolide (azithromycin) and a quinolone (levofloxacin) for his pneumonia. Moreover, he was taking amlodipine on a regular basis before his admission for his hypertension.

His blood analysis revealed neutrophilic leucocytosis and mild eosinophilia with no internal organ involvement. The cultures were negative. The biopsy was compatible with pustular psoriasis, and his subsequent patch test was positive for cefuroxime and negative for azithromycin. Our working diagnosis was as follows:

1. (Initial clinical impression): Acute generalized exanthematous pustulosis.
2. (Differential diagnoses): Pustular psoriasis, drug hypersensitivity syndrome and folliculitis.
3. (Analysis of drug exposure).
4. (Analysis of literature): AGEP is associated with both cefuroxime and azithromycin.
5. (Analysis of laboratory results): Neutrophilia, mild eosinophilia, no internal organ involvement, histopathology compatible with a pustular psoriasis and a positive patch test for cefuroxime.
6. (Prioritization of diagnoses): Cefuroxime-induced AGEP remained highly probable; pustular psoriasis possible, azithromycin-induced AGEP unlikely and drug hypersensitivity syndrome and folliculitis are almost excluded.

Hence, in this manner, with a little sincere effort and intelligent use of clinicopathologic parameters, one can easily set aside the probable drug, likely to be the inciting agent, in a particular case of ACDR. Although it is not as easy as it seems to identify a particular drug in a scenario in which a patient is taking multiple drugs, every effort should be made to identify it by reasoning and right approach.

## Management

We all know that once an offending drug is identified, it is easier to plan the management of ACDR as a common procedure. I will be highlighting the strategy in a nutshell.

The ultimate goal is always to discontinue the offending medication if possible. Individuals with drug eruptions are often very ill patients taking a large number of medications, many of which are essential for their survival. However, all nonessential medications should be limited. Once the offending drug has been identified, it should be promptly discontinued. The knowledge of the common eruption-inducing medications may help in identifying the offending drug. The decision whether to continue to administer a drug that is known or assumed to be the cause of a reaction will be influenced by the following factors:

1. Severity and probable course of the reaction
2. Disease for which the drug was prescribed
3. Ease or difficulty with which the reaction can be managed
4. Availability of chemically unrelated drugs with similar pharmacologic properties

Attention is given to ensure that any substituted drugs are not pharmacologically and/or chemically related to the suspect drug. For example, penicillin allergy reactions have cross-reactivity with cephalosporins, phenytoin hypersensitivity syndrome has cross-reactivity with phenobarbital and carbamazepine and sulfonamide reactions cross-react with other sulfa-containing drugs. In decisions regarding the discontinuation of a drug, one must consider the risk/benefit ratio of each drug.

- The therapy for most drug eruptions is mainly supportive and treatment depends on the specific typex of reaction. The therapy for exanthematous drug eruptions is supportive. First, the generation of antihistamines is used around the clock. Mild topical steroids (hydrocortisone or desonide) and moisturizing lotions are also used, especially during the late desquamative phase.
- Severe reactions, such as Steven Johnson syndrome, toxic epidermal necrolysis and hypersensitivity reactions, warrant hospital admission. Toxic epidermal necrolysis is best managed in a burn unit with special attention given to electrolyte balance and signs of secondary infection. Because adhesions can develop and result in blindness, ophthalmologic evaluation is mandatory. Further, there is mounting evidence that intravenous Ig (IVIG) may improve outcomes for TEN patients. Hypersensitivity syndrome - a systemic reaction characterized by fever, sore throat, skin rash and internal organ involvement - is potentially life threatening. The timely recognition of the syndrome and immediate discontinuation of the anticonvulsant or other offending drug is crucial. Patients may require liver transplantation if the drug is not discontinued in time. Treatment with systemic corticosteroids has been advocated.

## Conclusion

After a cutaneous drug eruption has been diagnosed and treated, clear information must be provided to the patient regarding his/her drug rash. Advise patients to carry a card or some other form of emergency identification in their wallets that lists drug allergies and/or intolerances, especially if they have had a severe reaction.

The names of the medication, potentially cross-reacting drugs and drugs that can be safely taken are an important part of the evaluation. The predisposition to some drug-induced eruptions may be genetic and family counseling is part of the care plan. This can be important especially in SJS, TEN, drug hypersensitivity syndromes and SSLRs. Finally; cutaneous drug reactions should be reported to the manufacturer and the regulator agency especially if the skin eruption is rare, serious or unexpected.

Adverse cutaneous drug reactions are distressing to both the patient and physician; when they are more effective and potent drugs are being developed, it is inevitable in modern day practice. However, it is incumbent on us as physicians to weigh the benefits and risks of each and every therapeutic decision carefully. We must be alert to potential adverse events and to recognize them early. However, it is better if we can prevent them from happening.

Besides, drug reactions are a common reason for litigation. Not warning a patient about potential adverse effects, prescribing a medicine to a previously sensitized patient or prescribing a related medication with cross-reactivity are the most common medicolegal pitfalls; therefore should not be ignored or taken lightly.
